# Neuronal Replacement as a Tool for Basal Ganglia Circuitry Repair: 40 Years in Perspective

**DOI:** 10.3389/fncel.2020.00146

**Published:** 2020-05-29

**Authors:** Anders Björklund, Malin Parmar

**Affiliations:** Developmental and Regenerative Neurobiology, Department of Experimental Medical Science, Wallenberg Neuroscience Center, Lund University, Lund, Sweden

**Keywords:** embryonic stem cells, induced pluripotent stem cells, regenerative medicine, striatum, substantia nigra, nigrostriatal pathway, dopamine

## Abstract

The ability of new neurons to promote repair of brain circuitry depends on their capacity to re-establish afferent and efferent connections with the host. In this review article, we give an overview of past and current efforts to restore damaged connectivity in the adult mammalian brain using implants of fetal neuroblasts or stem cell-derived neuronal precursors, with a focus on strategies aimed to repair damaged basal ganglia circuitry induced by lesions that mimic the pathology seen in humans affected by Parkinson’s or Huntington’s disease. Early work performed in rodents showed that neuroblasts obtained from striatal primordia or fetal ventral mesencephalon can become anatomically and functionally integrated into lesioned striatal and nigral circuitry, establish afferent and efferent connections with the lesioned host, and reverse the lesion-induced behavioral impairments. Recent progress in the generation of striatal and nigral progenitors from pluripotent stem cells have provided compelling evidence that they can survive and mature in the lesioned brain and re-establish afferent and efferent axonal connectivity with a remarkable degree of specificity. The studies of cell-based circuitry repair are now entering a new phase. The introduction of genetic and virus-based techniques for brain connectomics has opened entirely new possibilities for studies of graft-host integration and connectivity, and the access to more refined experimental techniques, such as chemo- and optogenetics, has provided new powerful tools to study the capacity of grafted neurons to impact the function of the host brain. Progress in this field will help to guide the efforts to develop therapeutic strategies for cell-based repair in Huntington’s and Parkinson’s disease and other neurodegenerative conditions involving damage to basal ganglia circuitry.

## Introduction

The idea that new cells—neurons, neuroblasts, or immature neural precursors—can be used to repair damaged neural circuitry in the brain goes back to the late 1970s. These early studies were performed in rodents and made use of neuroblasts obtained from the developing CNS. The experiments pursued in our lab here in Lund were focused on the use of tissue dissected from the developing ventral mesencephalon or fetal striatal primordia (i.e., the ganglionic eminences) to replace the lost nigral or striatal neurons and restore axonal connectivity in rats with damage to the basal ganglia circuitry, similar to that seen in patients with Parkinson’s or Huntington’s disease.

Neural transplantation is a classic approach in cold-blooded vertebrates, salamanders, fish, and frogs, that goes back to the early decades of the last century. Similar studies in mammals were initially unsuccessful due to shortcomings of the methods used at the time, and it was not until the 1970s, with the introduction of histochemical methods and modern tract-tracing techniques, that the effective tools for the study of survival and growth of neural tissue became available (for reviews of these early developments see Thompson and Björklund, 2015; Dunnett and Björklund, 2017). Subsequent progress has been critically dependent on the development of increasingly more refined and powerful techniques for studies of neuronal connectivity that has taken place over the last decades. The early studies used methods that allowed selective visualization of specific neuronal systems, defined by their transmitter content, or on the use of species-specific antibodies that allow immunohistochemical staining of, e.g., mouse, pig or human neurons and their axonal projections in the rodent brain or spinal cord. These methods were combined with classic anterograde or retrograde tracers injected into the grafted tissue or selected anatomical targets in the host brain. Decades later, the possibility to create transgenic animals and cell lines expressing fluorescent reporters, such as green fluorescent protein (GFP), provided a new set of powerful and versatile tools to trace axonal projections, making it possible to study the connectivity of neural grafts with a sensitivity and specificity that went beyond what had been possible with classic tract-tracing techniques.

Contrary to the prevailing notion at the time, the results obtained in these pioneering studies provided compelling evidence that new neurons can be anatomically integrated into damaged brain circuitry, and that neurons developing from implanted neuroblasts exhibit a remarkable capacity to recreate functional efferent and afferent connections with the damaged host brain. From the very beginning of these studies, the striatum, and its cortical, thalamic and brain stem connections, proved to be a very useful testbed for the development of this cell-based repair approach. There were tools available that allowed selective damage to components of this circuitry, and a battery of tests for striatum-related motor and cognitive behaviors, suitable for monitoring of behavioral impairment and recovery in lesioned rats and mice, had been developed. The two most commonly used brain lesion models—ablation of striatal projection neurons using excitotoxic lesions, induced by ibotenic acid (IBO) or quinolinic acid (QUIN), and damage to the nigrostriatal dopamine (DA) system using the 6-hydroxydopamine (6-OHDA) neurotoxin—were also attractive in that they replicated some of the key pathology seen in patients with Huntington’s and Parkinson’s disease.

During the last decade, the development of new genetic and viral techniques for the study of brain connectomics has opened new possibilities, and as a result, we are now entering a new phase in the study of cell-based brain repair, and at the same time, the approach using transplants of DA neurons derived from pluripotent stem cells (PSCs) is now entering the clinic in patients with Parkinson’s disease. The purpose of the present review is to summarize the progress made in this field, from the early days to the present, with a focus on the studies performed over the last four decades in our lab here in Lund, and discuss the possibilities and challenges for the development of cell-based therapies for brain circuitry repair.

## Basal Ganglia Circuitry and Motor Control

**Figure 1:**
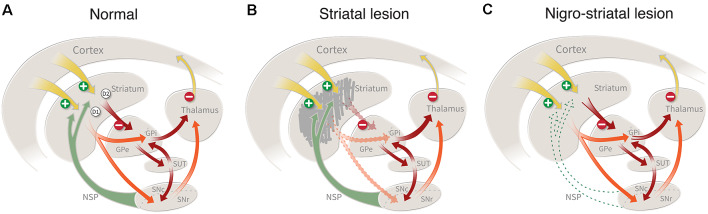
**(A)** Striatal connectivity comprises two major neuronal circuits: the cortico-striato-pallido-thalamic circuit and the cortico-striato-nigro-thalamic circuit, which in turn are interlinked by an important regulatory hub, the subthalamic nucleus. The striatal projection neurons (which constitute more than 90% of all neurons in the striatum) are of two types: the D1 receptor-bearing striatonigral neurons that innervate the internal globus and the pars reticulata of the substantia nigra, and the D2 receptor-bearing striatopallidal neurons which innervate the external globus pallidus. **(B)** Damage to the striatal projection neurons, caused by an intrastriatal injection of ibotenic acid (IBO) or quinolinic acid(QUIN), will disrupt these two circuits and result in a disinhibition of the downstream targets, the pallidum and the substantia nigra, pars reticulata. This lesion mimics the striatal damage seen in patients with Huntington’s disease. **(C)** Lesion of the nigrostriatal dopamine (DA) pathway, induced by the injection of 6-hydroxydopamine (6-OHDA) or MPTP, removes an important regulatory control, resulting in motor impairments similar to those seen on patients with Parkinson’s disease.

In rodents, striatal connectivity is organized in a way that is very well suited for the exploration of repair strategies. The striatum contains a well-defined mixture of short-axoned interneurons and long-axoned spiny projection neurons that link the striatum with three major downstream targets, the external and internal globus pallidus and the substantia nigra. As illustrated schematically in Figure 1A, the projection neurons (which constitute more than 90% of all neurons in the striatum) are of two subtypes: the D1 receptor-bearing striatonigral neurons that innervate the internal globus and the pars reticulata of the substantia nigra, and the D2 receptor-bearing striatopallidal neurons which innervate the external globus pallidus. These projections are strictly unilateral, which means that a lesion on one side will leave the contralateral side intact to serve as an internal control. The projection neurons receive inputs from local interneurons, as well as from four major extrastriatal sources: glutamatergic excitatory afferents from neocortex and thalamus, and modulatory afferents from dopaminergic neurons in the ventral midbrain and serotonergic neurons in the pontine raphe nuclei (for review see Silberberg and Bolam, 2015).

The striatum plays a central role in the planning and execution of movement, as well as in the acquisition of motor skills and habits (for recent reviews see Redgrave et al., 2010; Graybiel and Grafton, 2015). As illustrated in Figure 1, cortical control is channeled through the striatum and its principal downstream targets, external and internal pallidum, and substantia nigra. The cortico-striatal inputs are topographically organized, such that areas associated with the sensorimotor function project to the dorsolateral part of the striatum, associative inputs to the dorsomedial part, and limbic inputs to the ventromedial part (including nc. accumbens). Consistent with this anatomical arrangement the striatum can be subdivided into three parts that subserve different aspects of motor behavior: *a dorsolateral part* regulating habitual, automatic movements (corresponding to the post-commissural putamen in humans); *a dorsomedial part* mediating associative and goal-directed behaviors (corresponding the rostral putamen and caudate nucleus in humans); and *a ventromedial/nc accumbens part* involved in motivational and emotional behavior (corresponding to the ventral striatum in humans; Redgrave et al., 2010; Graybiel and Grafton, 2015). These subsectors of the cortico-striatal machinery are functionally interconnected: their outputs converge in the downstream targets, globus pallidus, and substantia nigra, and they interact in the execution of coordinated motor behavior.

According to the classic model shown in Figure 1A, the two subtypes of striatal projection neurons are proposed to exert opposing influences on motor function, such that the neurons projecting to the internal pallidum and substantia nigra, called the *direct pathway*, act to facilitate movement, and the neurons innervation the external pallidum, the *indirect pathway*, serve to inhibit movement (Albin et al., 1989; DeLong and Wichmann, 2015). Although the functional interactions between these two output pathways are more complex than suggested by this simplistic model (see Redgrave et al., 2010), it seems clear that the overall inhibitory control exerted by the GABAergic projection neurons over their downstream targets is a key element in the initiation and execution of movement. As a consequence, damage to the cortico-striato-pallidal circuit induced by ablation of the striatal projection neurons will result in a disinhibition of the affected pallidal and nigral target cells and impaired motor function (Figure 1B; Chevalier and Deniau, 1990; Redgrave et al., 2010).

The DA neurons of the substantia nigra and ventral tegmental area (VTA) provide important modulatory control of striatal function. Importantly, the DA neurons are not themselves part of the motor execution pathway—i.e., the striatopallidal and striatonigral loops—meaning that removal of the DA input (as seen in nigra-lesioned animals or PD patients) will leave this circuitry intact, but in a dysfunctional state, resulting in a hypokinetic syndrome characterized by a difficulty to initiate and perform movements (Figure 1C). The nigrostriatal pathway exhibits a general medial-to-lateral topography, and the part that is most severely affected in PD is the lateral portion, i.e., the part that projects to the dorsolateral (sensorimotor) part of the striatum (Kish et al., 1988). This pattern of DA neuron loss is consistent with the fact that it is the execution of automatic movements and motor habits that are most severely, and also early, affected in PD patients (Marsden, 1982; Rodriguez-Oroz et al., 2009). Consequently, restoration of striatal DA neurotransmission, by drugs or DA neuron transplants, is the main therapeutic strategy for the recovery of motor function in patients with PD.

## Reconstruction of Basal Ganglia Circuitry in Animals With Striatal Lesions

Ablation of the striatal projection neurons disrupts major pathways for the execution of functions initiated at the level of the cerebral cortex and causes impairment of sensorimotor, associative, or limbic behaviors. These impairments are caused by the removal of inhibitory control of functionally related downstream targets, the substantia nigra, and the external and internal pallidum (see above). Conceived in this way, the impairments seen in striatum-lesioned animals can be viewed as a “disconnection syndrome” caused by a disruption of the cortico-striato-pallido/nigro-thalamic circuits that subserve these functions. The cell-based repair strategies pursued in this model seek to restore connectivity and function in these execution pathways.

**Figure 2:**
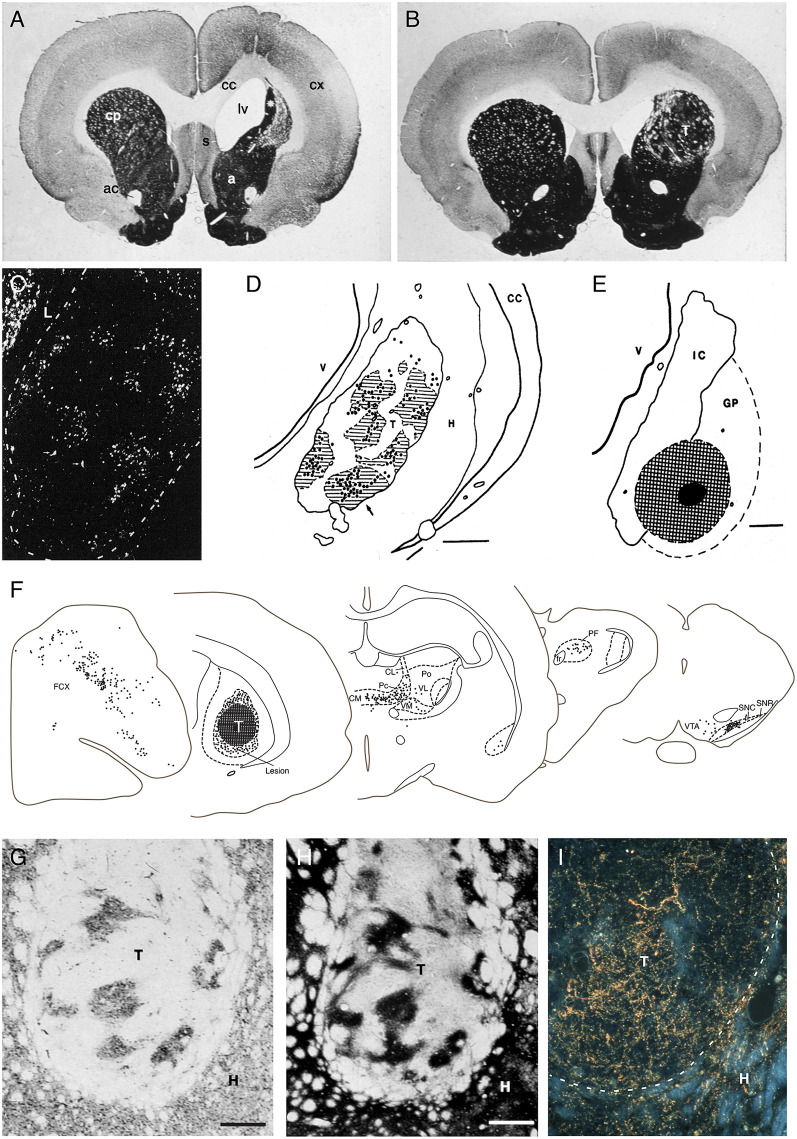
**(A,B)** The loss of striatal neurons, and the reduction in overall striatal volume, seen in the excitotoxin lesioned striatum (as shown in **A**), are largely restored by the transplanted fetal striatal primordium (**B**; AchE stain, 6 months survival. T = transplant). **(C–E)** Injection of the retrograde axonal tracer FluoroGold into the Globus pallidus (hatched area in **E**) results in the labeling of large numbers of DARPP-32+ striatal projection neurons in the graft (bright spots in **C**). The vast majority of these are located within the DARPP-32+ patches in the grafts (striped area in **D**). **(F)** Injection of a retrograde axonal tracer into the striatal graft (hatched area, T) reveal extensive afferent inputs from the same regions of the host brain that innervate the striatum in the intact brain, including the frontoparietal cortex (FCX), the intralaminar thalamic nuclei (CL, CM, PF, Pc, Po, VL, VM), and the substantia nigra (SNC), with a distribution that is closely similar to that seen in the intact animal. **(G–I)** The DARPP-32+ areas of the grafts **(G)** are densely innervated by TH+ axons from the host nigrostriatal pathway **(H)**, as well as from the host frontal cortex (labeled with the anterograde tracer PHA-L in **I**) adapted from Wictorin et al. (1989b) and Wictorin and Björklund (1989).

The excitotoxic lesion used in these studies causes a rapid neuron death followed by a gradual shrinkage of the striatal volume within the following weeks (Isacson et al., 1985). When applied unilaterally, as shown in Figure 2A, the lesion will cause motor asymmetry and a distinct impairment in contralateral paw use that can be monitored in the so-called staircase test, and when applied bilaterally the rats will develop a hyperactivity syndrome accompanied by pronounced cognitive and motivational impairments (for review see Dunnett et al., 2000). The cells used for grafting in this model are obtained from the fetal striatal primordia residing in the ganglionic eminence (GE). GE, however, is a heterogeneous structure that gives rise not only to neurons in the striatum but also to other areas. Thus, the lateral part of this structure, lateral ganglionic eminence (LGE), is the source of the striatal projection neurons (as well as interneurons destined for the olfactory bulb), and the medial part, the MGE, is the source of the striatal interneurons, and it contributes also to the formation of interneurons in the cortex, pallidum and other ventral forebrain regions.

### Development and Composition of the Striatal Grafts

In our studies, we have used GE cells obtained from 14 to 15 day old rat fetuses and injected them into the lesioned site as a crude cell suspension. These graft deposits grow and mature with a time-course that is fairly close to normal striatum: they expand 5- to 8-fold in size over the first 3 weeks and reach their final size and maturation after 2–3 months (Isacson et al., 1984, 1986; Labandeira-Garcia et al., 1991). At this time they have expanded to occupy a major part of the lesioned and neuron-depleted part of the host striatum (Figure 2B). Interestingly, the growth and development of the grafts depend on the conditions at the transplantation site: grafts placed in the non-lesioned striatum are initially (at 4 days) of the same size, but they fail to grow in size at longer survival times (Labandeira-Garcia et al., 1991).

Neurons expressing characteristic markers of striatal projection neurons and interneurons are distributed in patches throughout the graft tissue, suggesting that the grafted striatal primordia can continue their fetal development in their new location. Further studies have shown that the striatal component is derived from the LGE, while the non-striatal areas, which express neuronal markers characteristic of cerebral cortex and globus pallidus, is largely derived from the MGE (Graybiel et al., 1989; Wictorin et al., 1989b; Campbell et al., 1995; Olsson et al., 1995). Thus, the proportion of the grafts expressing striatal markers can be greatly enhanced, from 30% to 40% in whole GE grafts up to 80–90%, if the dissection of the GE is restricted to the dorsal part of the LGE (Pakzaban et al., 1993; Olsson et al., 1995).

The neuronal composition of the striatum-like areas is notably similar to that seen in the intact striatum. As in the intact striatum, the vast majority of the neurons (over 90%) are of the DARPP-32 positive medium spiny type, and the two major subtypes, the pre-proenkephalin (PPE) and pre-protachykinin (PPT) expressing neurons, which are the characteristic markers of the striatopallidal and striatonigral projection neurons, respectively, are present in similar proportions, around 50%, as in the host (Campbell et al., 1992, 1995; Liu et al., 1992). The interneuron population has not been fully characterized, but it has been shown to include the characteristic type of large-sized cholinergic (ChAT positive) interneurons, as well as various types of GABAergic (GAD positive) interneurons that are seen to make connections within the grafts (Roberts and Difiglia, 1988; Graybiel et al., 1989; Helm et al., 1992; Clarke and Dunnett, 1993).

### Afferent Host-to-Graft Connectivity

The anatomical integration of the GE grafts has been extensively investigated using a combination of classic anterograde and retrograde tracers. These studies show extensive afferent inputs from the same regions of the host brain that innervate the striatum in the intact brain, including the frontoparietal cortex, the intralaminar thalamic nuclei, the basolateral amygdala, substantia nigra and the dorsal raphe nucleus, with a distribution that is closely similar to that seen in the intact animal (Figures 2F–I; Wictorin et al., 1988, 1989a; Wictorin and Björklund, 1989; Labandeira-Garcia et al., 1991). Simultaneous injection of two retrograde tracers, rhodamine-labeled beads into the grafts and True Blue into the adjacent, spared striatum, showed that the vast majority, 60–100%, of the cells in thalamus, substantia nigra and dorsal raphe were double-labeled, indicating that the host inputs to the striatal grafts are derived from axons that remain in the neuron-depleted area, i.e., from the very same neurons that project to the area of the striatum was the graft is placed.

Ultrastructural studies have shown that the cortical and thalamic afferents from asymmetric synaptic contacts with the grafted striatal neurons. As in the intact striatum, the contacts are made on both dendritic spines and shafts with a predominance of spine synapses, although the relative proportion of spine synapses tends to be lower than in the normal striatum (Wictorin et al., 1989a; Xu et al., 1991; Clarke and Dunnett, 1993; Dunnett and Björklund, 2017). The nigral TH-positive afferents provide a dense innervation of the DARPP32-positive striatum-like patches (Figures 2G,H) and have been shown to make synaptic contacts onto dendrites and spines of medium spiny grafted neurons (Wictorin et al., 1988, 1989b). Interestingly the cortical and dopaminergic inputs were seen to converge onto the same spines and shafts of neurons projecting to the host globus pallidus, similar to the connectivity seen in the intact striatum (Clarke et al., 1988b; Clarke and Dunnett, 1993). The functionality of the cortical input is supported by electrophysiological recordings (Rutherford et al., 1987), and also by the use of the c-Fos expression as a cellular marker of stimulation-induced functional activity (Labandeira-Garcia and Guerra, 1994) showing prominent activation of c-Fos in the DARPP-32 positive neurons induced by stimulation of the frontal cortex. The density of c-Fos positive nuclei within the DARPP-32 positive striatum-like patches was around 60% of that seen in the intact striatum, suggesting that the host cortical inputs can exert a wide-spread control of the grafted striatal projection neurons.

### Efferent Graft-to-Host Connectivity

The efferent projections of the grafted neurons are most efficiently visualized in xenograft experiments where the mouse, pig, or human GE are transplanted to the lesioned rat striatum and visualized by immunostaining using species-specific antibodies. This allows the grafted cells and the graft-derived axonal projections to be traced with high sensitivity and in their entirety. The results obtained using this approach (Wictorin et al., 1991) reveal a remarkable specificity in the axonal outgrowth pattern. The axons grow exclusively in one direction, caudally, and are confined to a single bundle that extends along with the myelinated fiber bundles of the internal capsule, ramifying into branches of terminals within the globus pallidus. Studies using a combination of anterograde and retrograde axonal tracers have shown that the outgrowing axons are derived almost exclusively from the DARPP-32 positive GABAergic medium spiny neurons (Figures 2C–E; Wictorin et al., 1989c; Campbell et al., 1995) and that they make synaptic contacts with dendritic shafts and spines of the host pallidal neurons, similar to the ones made by the striatopallidal connection in the intact animal (Wictorin et al., 1989a). The axonal outgrowth from grafts of rat or mouse GE tissue does not reach beyond the globus pallidus, which is in contrast to the more extensive outgrowth obtained from grafts of fetal human or pig GE tissue which extends along the nigrostriatal pathway to the substantia nigra, indicating that the growth capacity of the striatal neurons reflects their size in the donor species, small in rodents but much larger in pigs and human (Wictorin et al., 1990; Isacson and Deacon, 1996).

### Dopaminergic Regulation of Graft Function

**Figure 3:**
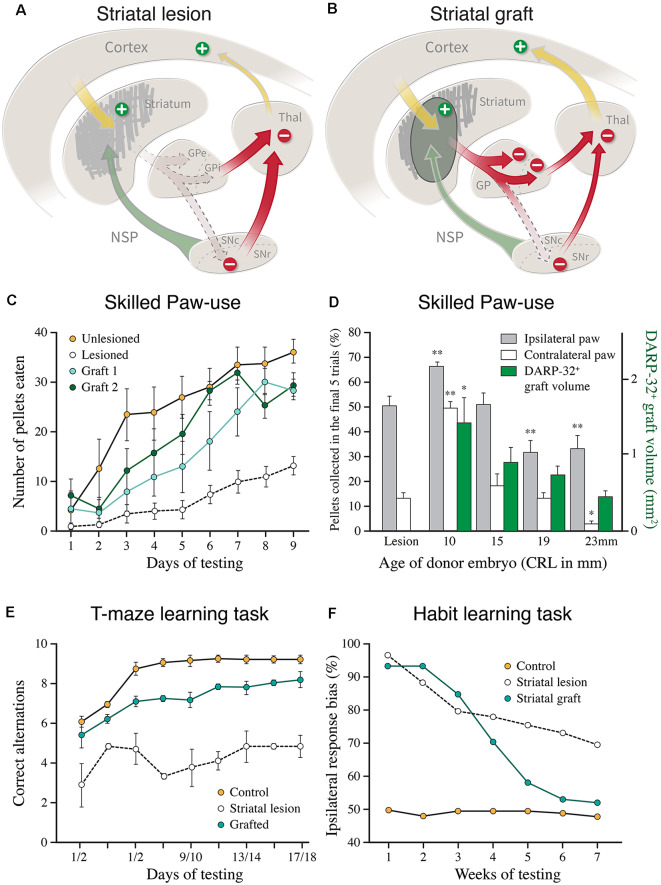
**(A,B)** Cartoons illustrating the disinhibitory effect of the excitotoxic striatal lesion on the downstream targets, globus pallidus (GPe and GPi), and substantia nigra pars reticulata (SNc), and the reversal of this effect induced by the striatal graft. **(C)** Recovery of skilled motor performance is the paw reaching test, as seen in two groups of lesioned and grafted rats, using grafts derived from the lateral ganglionic eminence (LGE) only (modified from Nakao et al., 1996). **(D)** Recovery of the use of the paw contralateral to the lesion and grafted side (open bars) is well correlated to the volume of the DARPP-32+ portion of the fetal GE grafts, obtained from the whole GE at different donor ages (green bars; modified from Fricker et al., 1997). **(E)** Graft-induced recovery in the performance of delayed alternation in the classic T-maze task in rats with bilateral striatal lesions and transplants (modified from Isacson et al., 1986). **(F)** Graft-induced recovery of habit learning in rats with unilateral striatal lesions and transplants. In this test, the grafted animals had to relearn the task over a similar period, 6–8 weeks, as seen in intact rats learning the same task for the first time (modified from Brasted et al., 1999). *p < 0.05, **p < 0.01.

These findings show that the key components of the cortico-striato-pallidal circuitry are re-established in the fetal GE grafts (Figures 3A,B). An additional essential component is the host dopaminergic input. Immunohistochemistry shows that the striatum-like regions of the GE grafts receive a dense synaptic innervation from the host substantia nigra that converges with the cortical input onto the dendrites and spines of the medium spiny neurons in the graft (Figures 2G,H; Wictorin et al., 1989b; Clarke and Dunnett, 1993).

Several lines of evidence suggest that the host DA innervation is functional and that it is likely to play the same regulatory role as in the intact striatum. One approach has been to use cellular markers of neuronal function, such as neuropeptide mRNA and c-Fos expression, to monitor the level of afferent dopaminergic control. The DA afferents are known to exert a differential regulation over the two major output pathways: inhibitory for the D2 receptor and PPEmRNA expressing striatopallidal neurons, and excitatory for the D1 receptor and PPTmRNA expressing striatonigral neurons (Figure 3B). The dopaminergic control of these two transcripts—down-regulation of PPEmRNA in the D2 neurons and up-regulation of PPTmRNA in the D1 neurons—is as efficient in the striatal grafts as in the normal striatum (Campbell et al., 1992; Liu et al., 1992). In further support, it has been shown that DA releasing drugs (amphetamine and cocaine), which are known to induce c-Fos expression selectively in the D1 bearing striatonigral neurons, are as effective in the grafted animals as in the intact striatum. This effect was abolished by the 6-OHDA lesion of the host nigrostriatal input (Liu et al., 1991; Mandel et al., 1992).

Together, these data show that the striatal efferent projections are re-established by the GE grafts and that they are under the control of the host DA system (Figures 3A,B). The sparse graft projection to the host substantia nigra, however, suggests that the functional effects obtained with rat fetal GE grafts are mediated primarily by their pallidal connection.

### Behavioral Evidence for Circuitry Repair

Graft-induced functional recovery has been observed in behavioral tasks at different levels of complexity: locomotor activity, skilled paw use, habit learning, and conditioned motivational behaviors (for review see Dunnett et al., 2000; Reddington et al., 2014). The ability of the GE grafts to restore function across this range of unconditioned and conditioned motor behaviors constitute the very best example, so far, of functional circuitry repair. As discussed above, the automatic and habitual motor behaviors (monitored in locomotor and paw use tests), habit learning, and conditioned motivational behaviors, are in the intact striatum mediated by different subsectors of the cortico-striatal machinery, converging onto their principal downstream targets, globus pallidus and substantia nigra. The integrated system converging onto the globus pallidus seems to be efficiently restored in the transplanted animals.

On the simplest level, the *locomotor hyperactivity* induced by bilateral striatal lesions can be described as the removal of a tonic inhibitory control of the striatal downstream targets, exerted by the GABAergic striatal projection neurons. This disinhibitory effect is supported by the observation that the activity of the external globus pallidus is markedly increased in striatum-lesioned rats (Isacson et al., 1984; Nakao et al., 1999). Viewed in this way, the normalization of locomotor activity seen in the GE transplanted rats is readily explained by the restitution of inhibitory control of the previously denervated pallidal neurons, mediated by the GABAergic striatopallidal connection formed by the DARPP-32 positive neurons in the grafts (see e.g., Isacson et al., 1986; Reading and Dunnett, 1995; Nakao et al., 1999).

The results obtained in the *skilled paw use test* (Figure 3C) are particularly interesting since the performance in this more complex test depends on the integrity of all major components of the striatal machinery, not only the striatum but also the cortico-striatal afferents and the nigral dopaminergic innervation (Whishaw et al., 1986). The recovery seen in the GE grafted rats in this test, therefore, is likely to reflect the functionality of the entire cortico-striato-pallidal circuit, as well as a functional dopaminergic control of this pathway (Montoya et al., 1990; Döbrössy and Dunnett, 2005; Klein et al., 2013). As illustrated in Figure 3D, the recovery is well correlated with the volume of DARRPP-32 positive striatal areas and the total number of DARPP-32 positive neurons in the grafts (Nakao et al., 1996; Fricker et al., 1997), and is not observed in rats with grafts of non-striatal tissue (Montoya et al., 1990). The quality of paw reaching, including all components of the movement, grasping and retrieval, was fully restored and equal to the performance of the intact controls (Klein et al., 2013).

The ability of the GE grafts to promote recovery in the paw reaching task is also interesting because it combines elements of motivation, learning, and acquisition of motor habits, as well as the execution of skilled motor behavior, i.e., the separate and interacting elements of skilled, complex movement that are subserved by different sectors of the striatum. It seems possible that a graft placed in the lateral sector of the striatum can interact with the ventromedial striatum/nucleus accumbens sector of the host (which is largely spared in these experiments), and that this interaction can take place at the level of the globus pallidus where the outputs of these parallel circuits converge (see above).

These findings indicate that the host cortical inputs play an important role in the graft-induced recovery of more complex motor behavior. This is further supported by studies using the *delayed alternation task* (Figure 3E), a classical learning task that is critically dependent on the prefrontal cortex and its connections with the medial striatum. Bilateral striatal lesions induce a marked and permanent impairment in this test, performed either in a T-maze (as shown in Figure 3E), or in an operant Skinner box. GE grafts have been shown to restore the ability of the grafted animals to perform this task (Isacson et al., 1986; Dunnett and White, 2006), and that this recovery is matched by extensive afferent input from the host prefrontal cortex (Dunnett and White, 2006).

Further evidence for circuit reconstruction, and support for the concept of “learning to use the transplant,” comes from studies on *habit learning*. The formation and maintenance of motor habits is a characteristic feature of striatal function (Knowlton et al., 1996; Packard and Knowlton, 2002). Studies in Steve Dunnetts lab have used a well-designed stimulus-response association task to explore the loss and recovery of this aspect of motor behavior in rats with unilateral striatal lesions and transplants (for a recent review see Dunnett and Björklund, 2017). These studies show that well-learned motor habits are lost in rats with striatal lesions, but that they can be relearned in the presence of a striatal graft (Figure 3F; Brasted et al., 1999, 2000). When tested several months after lesion and transplant the previously trained animals were as impaired as the lesion-only controls. The transplanted rats, however, were able to relearn the task over a similar period, 6–8 weeks, as seen in intact rats learning the same task for the first time, whereas the lesioned rats were unable to relearn even after extensive additional training. These data suggest that the GE grafts re-constitute a new habit-learning system that becomes functionally integrated into the lesioned host circuitry.

Taken together, these studies show that the GE grafts are remarkably effective in restoring both simple automatic and more complex motor behaviors of the type that normally depends on a well-functioning cortico-striato-pallidal circuitry, as well as a functional DA input. It is important, however, to keep in mind that the recovery in most cases is only partial and that the relearning seen in the delayed alternation and habit learning tasks is variable and level off, at a level that is below the optimal performance in the non-lesioned controls. This is perhaps not so surprising given that the fetal rodent GE grafts used in these studies restore only a relatively small fraction of the lost striatal neurons, and that their efferent connectivity is limited to the globus pallidus. i.e., the target of the indirect pathway (Figure 3B) while the prime target of the direct pathway, the pars reticulata of the substantia nigra, remains poorly innervated, although both major types of striatal projection neurons, the D1 and D2 expressing ones, are present in fairly equal numbers in these grafts (Campbell et al., 1992, 1995; Liu et al., 1992). Nakao et al. (1996) have estimated that the number of DARPP-32 positive projection neurons in well-functioning grafts amounts to around 30–40% of the lost neurons. This figure was obtained from grafts derived from the lateral part of the GE which yields a higher proportion of DARPP-32 positive tissue in the grafts, in this case about 60% of total graft volume. In most studies using grafts derived from the whole GE the DARPP-32 positive part is less than that, indicating that significant recovery also in complex motor behavior is obtained with grafts that replace as little as 20–30% of the lost striatal projection neurons. Despite these anatomical shortcomings, it is notable that the graft-induced recovery obtained in the best cases matches well that seen in non-lesioned controls.

Although the DARPP-32 positive striatal projection neurons constitute an essential component of a functional striatal graft, it remains unclear to what extent other complementary striatal neuron types, the GABAergicg, and cholinergic interneurons, in particular, may play a role. This remains to be explored, but it is interesting to note that the extent of recovery in the skilled paw use test (as reviewed above) appears to be similar in animals receiving transplants of the whole GE (Figure 3D; Fricker et al., 1997) or the lateral GE only (Figure 3C; Nakao et al., 1996). As mentioned earlier, these two graft types differ in their interneuron content: the medial part of the GE is the source of the striatal GABAergic and cholinergic interneurons, and as a result, these types of neurons are more or less lacking in the LGE grafts.

## Reconstruction of the Nigrostriatal Dopamine Pathway in Animal Models of PD

The nigrostriatal system has been in the focus of brain repair studies since the 1980s when the first exploratory intracerebral DA neuron grafting experiments were performed. It is experimentally attractive for several reasons: there are effective tools, the 6-OHDA and MPTP neurotoxins, that allow complete and selective lesions of the nigral DA projection, and there are accurate and relatively simple behavioral tests to monitor the effects of lesions and transplants. Moreover, the nigrostriatal pathway is strictly unilateral making it possible to use the non-lesioned side as an internal control. The motor impairments induced by lesions of the nigral DA system, which resemble the core motor symptoms in PD, are particularly interesting in the perspective of cell-based repair since they are due to loss of a component of the striatal circuitry that is regulatory, and thus not by itself part of the motor execution machinery. It is common to compare the role of the striatal DA input to the clutch in a car: it is needed to put the engine in gear, while the driving part of the motor is still intact. Thus, in DA lesioned animals as well as in PD patients, the cortico-striato-pallido/nigral circuitry that initiates and executes movements is intact, but its use is impaired or blocked due to the lack of the DA-mediated activating input, which regulates the threshold for the initiation of movement.

This modulatory role of the nigral DA neurons suggests the possibility to reverse the DA-lesion induced motor impairments, at least to some extent, using relatively simple approaches that restore DA neurotransmission. This is supported by the fact that L-DOPA therapy works well in PD patients, showing that tonic activation of striatal DA receptors is sufficient to provide significant symptomatic improvement, at least in the early stages of the disease.

The idea to use transplants of DA neurons to restore striatal DA neurotransmission goes back to the late 1970s. In these early studies, performed in 6-OHDA lesioned rats, fetal ventral mesencephalic (VM) tissue was transplanted either as a solid piece in direct contact with the striatum or as a cell suspension injected into the striatum. The grafted DA neuroblasts continued to develop in their new location and were seen to form an extensive functional axonal network in the surrounding host striatum. In their ectopic location, however, they were not in the correct position to reconstruct the entire nigrostriatal pathway and its full repertoire of afferent inputs (for review of these early studies see Winkler et al., 2000; Thompson and Björklund, 2012). More complete circuitry repair, as achieved in the striatal transplantation model (see above), is more of a challenge in the nigrostriatal system, since the axons of the nigral DA neurons extend over a relatively long distance along the nigrostriatal pathway to reach the striatum.

Full circuitry reconstruction requires that the grafted neurons are anatomically and functionally integrated into host neural circuitry. The presence of host afferent inputs to intra-striatal DA neuron transplants was initially studied using classical tract-tracing techniques. Although these studies have generated quite a lot of interesting information, it is only now, with the introduction of the monosynaptic rabies tracing technique (Wickersham et al., 2007), that we have access to a tool that can give us a more complete and also more detailed picture of the extent and identity of the afferent inputs to the grafts.

### Graft Composition

The fetal VM tissue commonly used in these studies contains the early progenitors of two major DA neuron types, the A9 neurons of the substantia nigra, and the A10 neurons that reside in the VTA and the medial part of the substantia nigra. In adult rodents, the two populations are present in roughly equal proportions (Björklund and Dunnett, 2007). In mature VM grafts the A9 neurons (Girk2+) are more numerous than the A10 (calbindin+) neurons, usually in the proportion of 2:1 (Grealish et al., 2010; Bye et al., 2012). The two types typically cluster together, with the A9 neurons located in the periphery and the A10 neurons residing in the graft core.

The DA neuron population, however, represents only a minority of the cells contained in mature VM grafts. The non-DA neuron population has not been fully characterized, but it is known that it contains both serotonin-, GABA-, enkephalin- and substance-P-containing neurons, as well as other types that cannot be readily identified based on neurochemical phenotype (for review see Thompson and Björklund, 2012). The relative proportion of serotonin neurons varies depending on the dissection of the VM tissue piece, from about 15–20% of the number of DA neurons as seen in standard VM preparations to about 50% of the DA component when dissection limit of the VM piece extends further caudally. The GABAergic component is likely to contain, in addition to local interneurons, two populations of projection neurons: the GABAergic neurons of the pars reticulata (normally projecting to thalamus and tectum) and the GABAergic neurons present in the VTA that constitute about 1/3 of the neurons in the VTA and are known to project widely to forebrain and brainstem targets (Taylor et al., 2014).

The standard VM grafts are also rich in cells with a glia-like morphology, some of which express the astrocyte marker GFAP. Although it is known from cell culture studies that astrocytes can provide essential trophic support for midbrain DA neurons (see e.g., Takeshima et al., 1994), experiments where the glial component is eliminated by cell sorting before grafting, or when fetal tissue form very early embryos before the onset of gliogenesis are used, suggest that the DA neurons survive and differentiate also in the virtual absence of glial cells (Thompson et al., 2006; Grealish et al., 2014).

### Target-Directed Axonal Outgrowth Patterns

**Figure 4:**
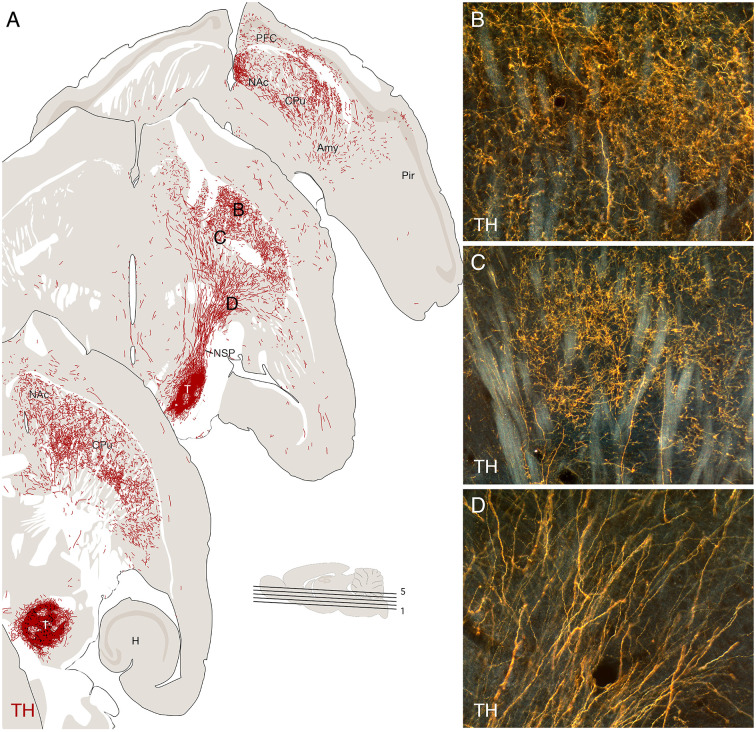
Re-establishment of the nigrostriatal pathway from a transplant of mouse fetal ventral mesencephalic (VM), implanted as a cell suspension in substantia nigra of a 6-OHDA lesioned mouse, 16 weeks post-grafting. The VM tissue was obtained from a transgenic Pitx3-green fluorescent protein (GFP) mouse allowing the outgrowing axons to be visualized using GFP immunostaining, as illustrated in the computer-assisted drawings derived from three horizontal sections in panel **(A)**. The micrographs in panels **(B–D)** are taken from the areas marked in panel **(A)**. Amy, amygdala; CPu, caudate-putamen; H, hippocampus; NAc, nc. Accumbens; NSP, nigrostriatal pathway; Pir, piriform cortex. Modified and redrawn from Thompson et al. (2009).

Regardless of their placement—in striatum or nigra—the graft-derived axonal projections are remarkably specific for their growth trajectory, as well as their innervated targets. The most striking examples come from observations made in rats with transplants of human VM tissue implanted at different sites along the nigrostriatal pathway (Wictorin et al., 1992; Grealish et al., 2014). Grafts placed either in the ventral midbrain or into the bundle project almost exclusively in the rostral direction and extend their axons along the course of the nigrostriatal pathway, toward the striatum, and along with the medial forebrain bundle (MFB) toward the normally DA-innervated areas of the limbic forebrain, matching well the distribution of DA projections in the intact brain (Figure 4).

The areas innervated by the VM grafts include territories that normally receive afferents from either the A9 neurons in the substantia nigra (i.e., dorsolateral caudate-putamen; CPu in Figure 4) or the A10 neurons in the VTA (i.e., nc. accumbens and prefrontal cortex; NAc and PFC in Figure 4). Both A9 and A10 neurons are present in the graft (see above) and there is evidence from tracing studies that the innervations derived from the two subtypes match the normal projection patterns, such that the A9 neurons innervate caudate-putamen and the A10 neurons cortical and limbic areas (Thompson et al., 2005; Grealish et al., 2010, 2014). These observations indicate the presence of axon guidance and target recognition mechanisms in the DA-denervated forebrain that can guide the growing axons to their appropriate targets and that these mechanisms are sufficiently specific and refined to distinguish between the A9 and A10 subtypes.

The demarcation of the innervated territories is remarkably precise: axons extending along the bundles of the internal capsule are seen to branch into fine-caliber beaded terminals as they pass the border between the globus pallidus and the caudate-putamen (Figures 4B–D), and in animals, with grafts placed in the caudate-putamen the graft-derived TH-positive innervation stops precisely at the border to the globus pallidus (Gage et al., 1983; Wictorin et al., 1992). Notably, studies comparing the performance of VM grafts in the intact and the DA-denervated striatum have shown that the density and extent of the graft-derived innervation are markedly increased in the absence of the intrinsic nigral input (Doucet et al., 1990; Thompson et al., 2005).

Although not studied in detail, it is clear that also the non-DA neurons in the VM grafts contribute to graft-host connectivity (Thompson et al., 2008; Grealish et al., 2014). Retrograde tracing studies indicate that these non-dopaminergic projections originate from GABAergic neurons contained in the grafts, and their wide-spread projection patterns, as revealed by species-specific antibodies in mouse-to-rat and human-to-rat grafts, suggest that they, at least in part, are derived from GABAergic neurons identical to the ones normally residing in the VTA and the pars reticulata of the substantia nigra (Thompson et al., 2008).

### Afferent Host-to-Graft Connectivity

From the outset, it has been assumed that DA neurons transplanted ectopically into the striatum are largely devoid of regulatory afferent inputs. In support of this idea, the early microdialysis studies showed that DA is released from intrastriatal VM grafts in an autoregulated fashion, suggesting that the grafted DA neurons maintain the capacity for regulated transmitter release even in the absence of afferent inputs (Zetterström et al., 1986; Strecker et al., 1987). A subsequent study in awake, behaving animals, however, showed that their activity as monitored by changes in DA release can be modulated during different types of behavior (Cenci et al., 1994). Although the behavior-related responses were lower in magnitude, and also less consistent than in intact rats, they suggest that the grafted neurons are reached by physiologically relevant inputs from the host brain. This finding is in line with electrophysiological studies showing that the majority of intrastriatal grafted DA neurons maintain their firing properties, including pacemaker activity, and that they show electrophysiological responses following stimulation of either host striatum or cortex (Fisher et al., 1991; Sorensen et al., 2005).

Anatomical data on the extent of host afferent inputs to fetal VM grafts are very sparse and limited to a single tract-tracing study (Doucet et al., 1989). As discussed further below, more recent studies performed using the more powerful monosynaptic rabies tracing method have shown abundant host inputs to human embryonic stem cell (hESC)-derived DA neuron grafts placed either in the striatum or the ventral midbrain (Grealish et al., 2015; Cardoso et al., 2018; Adler et al., 2019). For technical reasons, it has so far not been possible to perform similar rabies-based tracing studies on fetal VM grafts. The Doucet et al.’s (1989) study, using Phaseolus Vulgaris Leucoagglutinin (PHA-L) as an anterograde tracer, provides evidence for a fairly rich synaptic innervation from the host frontal cortex, as well as a limited serotonergic input to intrastriatal VM grafts, but other potential sources of afferents were not explored in this study. Nevertheless, the data we have from rabies tracing studies of hESC-derived DA neuron grafts, as discussed below, point to the capacity of grafted DA neurons to become extensively integrated into host circuitry.

### Restoration of DA Neurotransmission

Studies performed during the 1980s and 1990s had shown that the intrastriatal implanted nigral DA neurons are actively secreting their transmitter, and that part of this release may take place at morphologically normal synaptic sites. More direct evidence that graft-derived DA release is functional was obtained in studies using quantitative receptor-ligand binding techniques and *in situ* hybridization histochemistry. Lesions of the nigrostriatal DA pathway or blockade of striatal DA neurotransmission are known to lead to long-lasting postsynaptic modifications in the striatal target neurons. One such modification is the denervation-induced upregulation of postsynaptic DA receptors located on the striatal target neurons. This receptor supersensitivity is normalized by DA neuron grafts, most pronounced for the D2 receptors (Dawson et al., 1991; Rioux et al., 1991; Chritin et al., 1992; Savasta et al., 1992). This is consistent with the ability of DA neuron grafts to reduce turning behavior in unilateral 6-OHDA lesioned rats in response to either D1 or D2 (or mixed) receptor agonists. Rioux et al. (1991) reported parallel reductions in D1 and D2 agonist-induced turning and striatal receptor ligand-binding in rats with long-term surviving fetal VM grafts, suggesting that these parameters are closely linked.

The long-lasting functional changes seen in response to dopaminergic denervation include an increase in GABA turnover, accompanied by an increase in glutamic acid decarboxylase (GAD) enzyme activity and GAD mRNA expression in the DA-denervated striatum. Similarly, the enkephalin neurons, which constitute a subset of the D2 expressing GABAergic projection neurons projecting to the globus pallidus, express increased peptide levels as well as increased proenkephalin mRNA. This is maintained over a long time indicating that enkephalin synthesis is permanently increased in the striatopallidal projection neurons after removal of the striatal DA afferents. These changes are completely reversed by intrastriatal nigral transplants, thus providing further support that the grafted nigral neurons can restore normal inhibitory control over the host striatopallidal projection neurons (Cenci et al., 1993, 1997; Winkler et al., 2003).

The substance P containing striatonigral neurons respond to DA denervation in the opposite direction, i.e., by a reduction in peptide levels and RNA message. These effects are consistent with the current view that the two major subsets of striatal projection neurons are differentially regulated by the host nigral DA afferents, such that the striatopallidal enkephalin neurons are tonically inhibited and the striatonigral substance P neurons tonically activated by the DA input. In behaviorally functional grafts (as assessed by the rotation test) the effect on substance P synthesis is partial and restricted to the area receiving a dense DA input, while the increased enkephalin synthesis is fully normalized also outside the graft-reinnervated area, indicating that DA released from the graft-derived terminal network can reach functional levels over areas that extend well beyond that covered by the outgrowing fibers (Cenci et al., 1993; Strömberg et al., 2000).

Taken together, these findings point to two complementary modes of action: the diffuse release of DA by so-called volume transmission, acting primarily on the high-affinity D2 receptors, and regulated DA release at specialized synaptic sites involving both D1 and D2 receptors. As discussed further below, both mechanisms are likely needed to obtain the full extent of graft-induced behavioral recovery.

### Behavioral Correlates of Circuitry Repair

**Figure 5:**
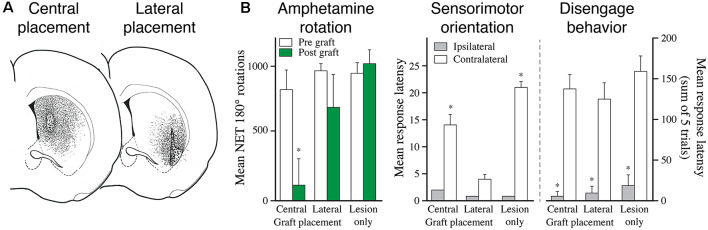
The functional effect depends on the area of the striatum reinnervated by the fetal DA neuron transplants. **(A)** The reinnervation obtained from VM cell suspension grafts placed in the central or lateral part of the striatum is restricted to the area surrounding the graft deposits. **(B)** Grafts reinnervating the central vs. lateral striatum, as shown in panel **(A)**, have markedly different effects on behavior: the centrally placed grafts abolish amphetamine-induced rotation but have little effect on sensorimotor behavior. The laterally placed grafts, by contrast, has little effect on amphetamine rotation but is highly efficient in restoring sensorimotor behavior. The more complex version of the task, called diseagage behavior, remains unaffected by these transplants but is well restored in animals with more wide-spread reinnervation of the striatal complex, as shown in Figure 6. Modified and redrawn from Mandel et al. (1990). *p < 0.01.

Drug-induced rotation is the most commonly used test to assess DA neuron function. In this test, an active turning behavior is induced in animals with a unilateral lesion of the nigrostriatal pathway using either the DA releasing drug, amphetamine, or the D1/D2 receptor agonist, apomorphine. In both cases, the functional recovery obtained with intrastriatal or intranigral VM grafts is readily explained by tonic activation of DA receptors mediated by volume transmission. Indeed, Savasta et al. (1992) have shown that the recovery in the agonist-induced rotation is well correlated with the normalization of D2 receptor binding in the grafted striatum and that this normalization involves also the non-reinnervated areas. As reviewed elsewhere (Björklund and Dunnett, 2019), full recovery in the drug-induced rotation is obtained with transplants that contain less than 500 DA neurons (i.e., about 4% of the normal number of nigral DA neurons in the rat) and restore as little as 5% of the normal striatal DA content. These transplants are too small to induce recovery in the standard tests of spontaneous motor or sensorimotor behavior. Significant recovery in these tests is seen only in animals with transplants rich in DA neurons that provide more extensive striatal reinnervation, suggesting that the extent of reinnervation, i.e., functional synaptic inputs, play an important role. This is further supported by extracellular recordings showing that the increased striatal neuron activity seen in the lesioned rats have normalized in the graft reinnervated area, but not in the non-reinnervated part (Di Loreto et al., 1996; Strömberg et al., 2000). In line with these observations, it has been shown that the functional impact depends on the area innervated by the grafted DA neurons. As illustrated in Figure 5, it has been shown that the recovery of individual components of the DA lesion syndrome is determined by the subregion reinnervated by the transplant; dorsomedial vs. ventrolateral striatum in the rat (Mandel et al., 1990), and caudate vs. putamen in the marmoset (Annett et al., 1995).

The synaptic contacts formed by the grafted DA neurons on the host striatal projection neurons resemble, in part, those present in the intact striatum, although their relative distribution is different—less frequent on dendrites and more abundant on the neuronal perikarya (Freund et al., 1985; Mahalik et al., 1985; Clarke et al., 1988a). The synapses made on dendritic spines, which constitutes about 40% of all TH-positive synapses formed, resemble those seen in normal animals, both in that they make contacts with spine necks and in that they are associated with an asymmetric TH-negative synapse contacting the spine head. This resembles the arrangement seen in the intact striatum where dopaminergic and cortical glutamatergic inputs converge onto the same spines (Smith and Bolam, 1990; Xu et al., 2012), suggesting the creation of a local microcircuitry allowing the graft-derived DA innervation to interact with the host corticostriatal input.

In a more recent study, Rylander et al. (2013) have added an interesting dimension to these early findings, showing that a region-specific and DA-dependent long-term potentiation (LTP), which is abolished in 6-OHDA lesioned rats, is fully restored in the grafted animals. This form of synaptic plasticity is NMDA receptor (i.e., glutamate) dependent and mediated by D1 receptors. Interestingly, this effect was limited to the most densely reinnervated (ventrolateral) part of the striatum where the TH-positive innervation density was restored to over 60%. This is consistent with observations in animals with partial 6-OHDA lesions showing that induction of LTP in the striatal projection neurons is critically dependent on a rich DA innervation (Paille et al., 2010). It seems possible that this mechanism is involved in the establishment and maintenance of stimulus-response habits, which is a characteristic feature of the dorsolateral sector of the striatum (Redgrave et al., 2010; see above). In line with this idea, Dowd and Dunnett (2004) have shown that DA neuron transplants targeting this striatal subregion are efficient in restoring learned, goal-directed behavior, assessed in a task that involves the selection, initiation, and execution of conditioned responses on either side of rat’s head, a habit learning task that is likely to depend on the local interaction between dopaminergic and glutaminergic (i.e., cortical) afferents.

### Limitations to the Functionality of the Intrastriatal DA Neuron Grafts

**Figure 6:**
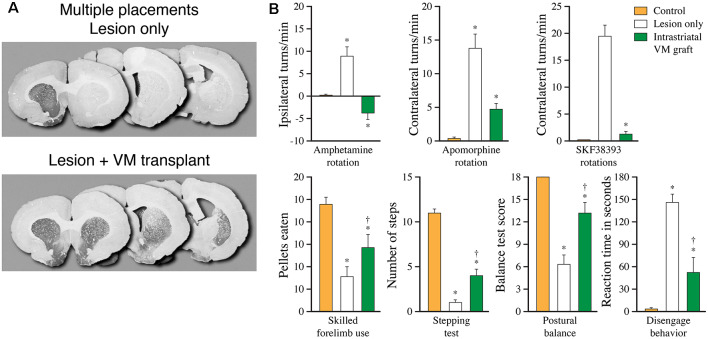
More complete recovery can be obtained by spreading the graft tissue over multiple implantation sites. **(A)** Extent of graft induced reinnervation obtained with fetal VM grafts spread over seven injection sites distributed over the entire striatum, including the nc. accumbens, 10 months post-grafting. **(B)** In these animals, significant functional recovery is seen in a broad range of drug-induced and spontaneous motor tests, but remains incomplete in most of the tests Data compiled from Winkler et al. (1999). *Different from Control at *p* < 0.05; ^†^different from Lesion only at *p* < 0.05.

Available data indicate that a striatal DA reinnervation in the order of 20–30% of normal is necessary to obtain measurable improvements in tests of spontaneous motor behavior, such a forelimb stepping, and paw-use. As illustrated in Figure 6, such a more wide-spread reinnervation can be obtained with VM tissue implanted at multiple sites in the striatum. Using multiple graft placements it is possible to restore striatal DA innervation density up to 60–70% of normal throughout the striatum, but even in such cases the recovery of more complex motor behavior is incomplete (Winkler et al., 1999). One factor that may play a role is the extent of the graft-derived reinnervation outside the caudate-putamen. The innervation generated by intrastriatal DA neuron grafts in the MFB lesioned rats (the lesion most commonly used in these studies) is confined to the dorsal striatum, while other DA-innervated forebrain areas, including nc. accumbens, olfactory tubercle, and the frontal and cingulate cortex, remain denervated. Indeed, in rats with partial 6-OHDA lesions where the projections to limbic and cortical areas are left intact, it has been shown that the magnitude of graft-induced recovery is more pronounced as assessed in the stepping and paw-use tests. Importantly, when the lesion, in a second step, was extended to remove the spared limbic and cortical projection is fully recovered animals, the graft-induced improvement in the two tests was partially lost (Kirik et al., 2001; Breysse et al., 2007). These data suggest that the extent of denervation outside the dorsal striatum will have an impact on the functional outcome, not only in the experimental setting but also in grafted PD patients. In support of this idea, Piccini et al. (2005) have reported that the best functional outcome in grafted PD patients is seen in subjects where the DA innervation in areas outside the grafted region is well preserved, as determined by FluoroDopa PET.

The most obvious limitation to the functionality of the intrastriatal grafts, however, is their ectopic location. As discussed further below, it has been shown that hESC-derived DA neuron grafts receive abundant host afferent inputs even in this ectopic location (Grealish et al., 2014; Adler et al., 2019). Full circuitry reconstruction, however, will require that the cells are placed in their normal location, i.e., the ventral midbrain. Progress in this field has been hampered by technical problems. In the early studies using rat fetal DA neurons grafted to the substantia nigra (Nikkhah et al., 1994; Winkler et al., 1999) no or very limited axonal growth was observed along the nigrostriatal pathway. From these studies, it became clear that fetal *rat* DA neurons do not have the growth capacity, or do not grow for a sufficiently long time, to reach the striatum in adult rats (although they manage to do so in neonatal rats; Bentlage et al., 1999). Fetal *mouse* DA neurons, by contrast, grow axons efficiently along the nigrostriatal pathway in adult mice (see Figure 4), but fail to do so in adult rats (Gaillard et al., 2009; Thompson et al., 2009). Nevertheless, it is possible to achieve the full reconstruction of the nigrostriatal pathway in rats using DA neurons of either human or porcine origin, i.e., neurons that intrinsically possess greater growth capacity and extend their growing axons over a longer period (Wictorin et al., 1989b; Isacson et al., 1995; Grealish et al., 2014).

The extent and origins of afferent inputs to intranigral grafts of fetal VM tissue have so far not been explored, and the functional impact of intranigral VM grafts has been limited to a single study in the mouse showing recovery in the amphetamine rotation test (Thompson et al., 2009). With the current emphasis on cells derived from pluripotent human stem cells (hPSCs), work along this line is likely to be pursued in studies using hPSCs rather than fetal VM tissue grafts. With the introduction of the monosynaptic rabies tracing technique, we have now access to a tool that can give us a more complete and also more detailed picture of the regulatory afferent inputs to intranigral DA neuron grafts.

## Studies Using Cells Derived From Human PSCs

The studies reviewed in the previous sections were mostly performed during the last decades of the past century. They were all based on the use of cells from the fetal brain, and the clinical trials in PD and HD patients have also been based on the use of fetal tissue. The experience gained from these trials has been highly valuable and provided important proof-of-principle that the neuronal replacement approach can work, at least in the case of PD. However, the use of fetal tissues for transplantation in patients is both ethically and practically problematic—they are difficult to obtain in sufficient numbers, impossible to standardize and quality control, and cannot be scaled up for routine clinical use. Further progress in this field, therefore, is critically dependent on the development of a scalable cell source specifically produced for use in patients. The discovery and derivation of hESCs in 1998 (Thomson et al., 1998) and the subsequent generation of induced pluripotent stem cells (iPSCs) in 2006 (Takahashi and Yamanaka, 2006) have revolutionized the field and provided new powerful tools for the derivation of virtually any cell type in the body, including the ones relevant for basal ganglia repair.

### Generation of Midbrain DA and Striatal Projection Neurons From Human PSCs

The development of protocols for the generation of midbrain DA (mDA) neurons and striatal projection neurons from hPSCs have progressed in parallel. The first attempts to generate mDA neurons from hESCs were based on protocols developed for mouse ESCs (see e.g., Kawasaki et al., 2000; Kim et al., 2002). Although they generated relatively large numbers of TH-expressing neurons *in vitro*, their midbrain properties were not clear, and they performed poorly after grafting. It is known from early experimental work that fully functional DA neuron transplants need to be of the midbrain A9 and A10 type. Thus, DA neurons of other phenotypes (hypothalamic or forebrain) do not show the ability to reinnervate the striatum in a target-specific manner, a property that is necessary for behavioral recovery.

An important breakthrough came in 2007–2008 with the discovery of the midbrain floor plate cell as the unique cellular progenitor of mDA neurons (Ono et al., 2007; Bonilla et al., 2008), which led to the development of protocols for the generation of floorplate cells (Fasano et al., 2010) and subsequently to DA neurons with an authentic midbrain phenotype (Cooper et al., 2010; Kriks et al., 2011; Kirkeby et al., 2012). These floorplate-derived cells express specific markers of mDA progenitors *in vitro* and their performance—survival, growth, and function—after transplantation in rodent and primate PD models matches very well that seen with authentic fetal mDA neurons (see e.g., Sundberg et al., 2013; Grealish et al., 2014; Chen et al., 2016; Kikuchi et al., 2017). With further refinement and transfer to production under GMP-compliant conditions completed, these cell products are now ready for use in patient trials (Kirkeby et al., 2017b; Studer, 2017; Takahashi, 2017).

The development of protocols for the generation of hPSC-derived striatal projection neurons is based on the same principle, i.e., to mimic the endogenous developmental process that takes place in the fetal ganglionic eminence as closely as possible (for review see Li and Rosser, 2017). In the most efficient protocols published so far striatal projection neurons, characterized by their DARPP-32 expression in combination with CTIP2 and/or GABA, are generated by modulation of the Sonic Hedgehog (SHH) and/or WNT signaling pathways (Ma et al., 2012; Delli Carri et al., 2013; Nicoleau et al., 2013; Adil et al., 2018; Wu et al., 2018). In an interesting alternative approach, Arber et al. (2015) have replaced the ventralizing factor SSH with a lateralizing factor, Activin A, to obtain cells with a striatal projection neuron fate.

The hESC-derived striatal progenitors survive and mature after transplantation to the lesioned rat or mouse striatum. The cells proliferate over the first weeks and as a result, the grafts expand in size to compensate for the striatal projection neuron cell loss caused by the excitotoxic lesion. Continued cell proliferation and signs of graft overgrowth have been an issue in some cases (Delli Carri et al., 2013; Nicoleau et al., 2013) suggesting that these graft preparations contain immature cells that fail to terminally differentiate. The percentage of DARPP-32+/CTIP2+ neurons generated in these protocols vary between 20 and 60%. Also, the grafts have been shown to contain other neuronal types, including GABAergic interneurons (Besusso et al., 2020), and in one case also a significant component (27%) of GFAP-positive astrocytes (Adil et al., 2018).

The expression pattern of the DARPP-23 positive cells indicates that they are striatal projection neurons of the type normally generated by the lateral ganglionic eminence (LGE). This is further supported by their ability to establish axonal connections with downstream striatal targets, including globus pallidus and substantia nigra, accompanied by a gradual improvement in measures of sensorimotor performance on the side opposite to the excitotoxic lesion and transplantation (Ma et al., 2012; Adil et al., 2018; Besusso et al., 2020). Although the extent and functionality of the graft-derived connectivity need to be explored in greater detail, the recent study by Besusso et al. (2020), using a combination of immunohistochemistry and monosynaptic rabies virus tracing, has provided initial evidence that the hESC-derived striatal grafts can become well integrated into the lesioned host striatal circuitry.

The extent of functional recovery seen in studies using fetal GE grafts, as reviewed above, has been mostly obtained with grafts that contain precursors from both the lateral and medial parts of the ganglionic eminence. While the striatal projection neurons normally arise from the LGE, the MGE is the source of the complementary populations of GABAergic and cholinergic interneurons, and as a result, the fetal GE tissue grafts contain also these types of interneurons. Although striatal projection neurons are an essential component, a fully functional striatal transplant is likely to contain a complement of regulatory interneurons, and possibly also supportive glial cells, that can form a functional unit with the capacity to replace the damaged host striatum. The optimal composition of such grafts, mimicking the fetal GE graft preparations, has yet to be experimentally determined.

### Integration and Function of hESC-Derived DA Neuron Transplants

The graft-host connectivity of hESC-derived mDA neurons generated in the different versions of the floorplate protocols have been studied in a xenograft setting, either in 6-OHDA lesioned rats and mice in combination with immunosuppressive treatment, or immunodeficient rats and mice (without the need of immunosuppression), after transplantation either to the striatum (Kriks et al., 2011; Sundberg et al., 2013; Grealish et al., 2014; Steinbeck et al., 2015; Chen et al., 2016; Niclis et al., 2017) or to the substantia nigra (Grealish et al., 2014; Cardoso et al., 2018; Adler et al., 2019). Similar results have also been obtained in MPTP-lesioned monkeys using hiPSC-derived, or primate iPSC-derived, DA neurons (Sundberg et al., 2013; Kikuchi et al., 2017). The performance of hESC-derived mDA neurons has been possible to compare head-to-head with that of the authentic mDA neuroblasts obtained from fetal human VM, showing that their survival, growth, and function, as well as their efferent connectivity pattern, match each other very closely (Grealish et al., 2014; Cardoso et al., 2018).

**Figure 7:**
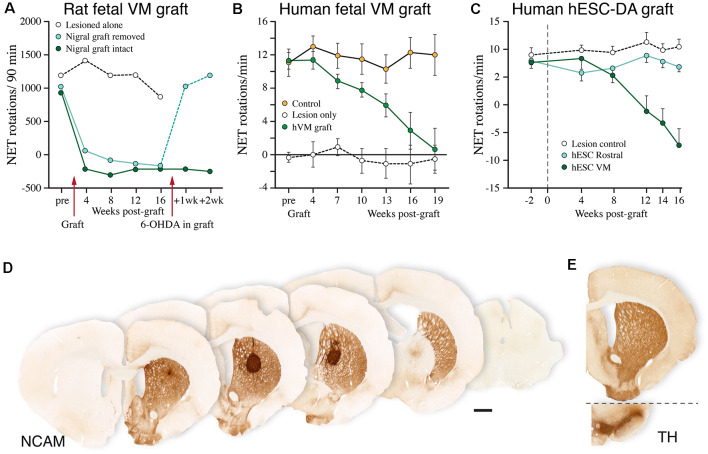
Performance of human fetal and human embryonic stem cell (hESC)-derived DA neurons grafted to the striatum in the rat 6-OHDA lesion model. **(A–C)** Time-course of functional recovery in the amphetamine rotation test obtained with fetal rat VM transplants **(A)**, human fetal VM transplants **(B)**, and hESC-derived DA neuron transplants **(C)**. The time-course of recovery is notably similar for the fetal and hESC-derived human DA neurons, but much slower than that seen with rat DA neurons. In the experiment shown in panel **(A)**, the original functional deficit returned within a week after the graft had been removed with a second 6-OHDA lesion. **(D)** A single deposit of hESC-derived DA neurons is sufficient to reinnervate the entire striatum in the rat PD model, as visualized using a human-specific NCAM antibody. The graft-derived innervation pattern in **(D)** is notably similar to the distribution of the endogenous TH-positive innervation, as shown in (**E**; adapted from Nolbrant et al., 2017). Data compiled from Dunnett et al. (1988; **A**), Lelos et al. (2016; **B**), and Kirkeby et al. (2012; **C**).

Transplants of human fetal or hPSC-derived mDA neurons mature slowly. As shown in Figures 7B,C, it takes, in rats, about 4–5 months before the connections become fully functional (compared to about 1 month in transplants of rat fetal VM, see Figure 7A), and it takes even longer, up to a year, in MPTP-lesioned monkeys where the growth distances are considerably larger (Kriks et al., 2011; Kirkeby et al., 2012; Sundberg et al., 2013; Lelos et al., 2016; Kikuchi et al., 2017). The axonal growth patterns from these transplants are highly specific and include innervation of developmentally appropriate targets of both A9 and A10 DA neurons (Figures 7D,E), indicating that the mDA neurons generated in the currently used floorplate-based protocols include the two major mDA neuron subtypes in proportions similar to the fetal VM grafts (Grealish et al., 2014; Niclis et al., 2017; Cardoso et al., 2018). This is further supported by the observation that hESC-derived mDA neurons transplanted to the substantia nigra have the same capacity to extend axons along the nigrostriatal pathway and the MFB as the fetal VM grafts, and the same ability to reinnervate the appropriate A9 and A10 targets. Axonal tracing using human-specific antibodies (hNCAM) in combination with TH immunohistochemistry (Grealish et al., 2014), or use of a Pitx3-GFP expressing hES cell line (Niclis et al., 2017), has shown that the projections are derived from both DA and non-DA neurons. As in fetal VM grafts (see above) the widespread projections of the non-DA neuron component suggest that they are generated by the GABAergic neurons normally present in the VM, above all the GABAergic component of the VTA and the pars reticulata of the substantia nigra.

**Figure 8:**
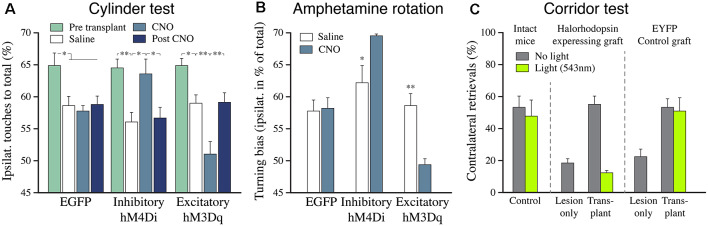
The functional impact of hESC-derived DA neuron grafts is tunable using chemo-and optogenetics. **(A)** The recovery of paw use in the cylinder test (contralateral to the 6-OHDA lesion) is abolished when the activity of the grafted DA neurons is inhibited by CNO (hM4Di), and it is further potentiated when the activity is increased by CNO (hM3Dq). **(B)** The effect of inhibition and activation of the grafted human DA neurons is similar in the amphetamine rotation test. The 6-OHDA-induced ipsilateral turning bias is abolished by the grafted DA neurons in all three groups (open bars). Activation of the inhibitory DREADD blocks the graft effect, seen as induction of an ipsilateral turning bias (similar to what is seen in lesioned controls), and activation of the excitatory DREADD potentiates the graft effect, seen as induction of turning in the direction away from the transplant. **(C)** The graft-induced recovery in sensorimotor performance seen in the corridor test (gray bars) is completely blocked when the activity of the grafted DA neurons is inhibited by light (green bars). Data in panels **(A,B)** are redrawn from Chen et al. (2016), data in panel **(C)** redrawn from Steinbeck et al. (2015). *p < 0.05, **p < 0.01.

The functionality of the hPSC-derived mDA neuron grafts is further supported by experiments where the activity of the grafted cells is modulated using either optogenetics (Steinbeck et al., 2015) or chemogenetics (DREADDs; Chen et al., 2016). In these studies, which are summarized in Figure 8, the recovered motor performance was completely reversed by optogenetic or chemogenetic inhibition of the hPSC-derived mDA neurons, and in the Chen et al.’s (2016) study it was shown that the graft-induced motor improvement could be further enhanced by DREADD-induced stimulation of mDA neuron function. Using electrophysiological recording in slices they moved on to show that these effects were synaptically mediated *via* DA receptor activation and that the grafted neurons modulate host glutamatergic transmission onto striatal projection neurons in a manner that is reminiscent of DA neurotransmission in the intact striatum.

These studies have been performed on grafts placed in the striatum, i.e., that same ectopic location as used in the clinical trials. It is commonly assumed that the functional impact of ectopically placed DA neuron grafts is mediated by an autoregulated, tonic activity and that they may not need access to regulatory afferent inputs that are available to the mDA neurons in their normal location in the midbrain. Recent studies using monosynaptic rabies tracing have challenged this view, showing that intrastriatal hESC-derived dopaminergic grafts receive synaptic inputs from subtypes of cortical, striatal and pallidal neurons that are known to regulate the function of the endogenous nigral DA neurons (Grealish et al., 2015; Adler et al., 2019). These regulatory inputs are known to send collateral within the striatum, which makes it possible for the intrastriatal grafts to receive inputs from functionally appropriate subtypes of excitatory (cortical) and inhibitory (striatal and pallidal) neurons in the host. Indeed, in a dual tracer approach, Adler et al. (2019) could show that the host synaptic inputs to intrastriatal grafts are derived, at least in part, from the very same cortical, striatal and pallidal neurons that innervate the host substantia nigra.

## Future Perspectives

The extent of graft-host connectivity obtained with intrastriatal grafts of fetal GE tissue is an intriguing example that more complete functional circuitry repair is possible to achieve in the lesioned rodent brain. As discussed above, the GE grafts are remarkably effective in restoring both simple automatic and more complex motor behaviors of the type that normally depends on a well functioning cortico-striato-pallidal circuitry, as well as a functional DA input. In most of the behavioral tasks used in these studies, however, the recovery seen with grafts of rodent GE tissue is only partial and obtained with grafts that replace as little as 20–30% of the lost striatal projection neurons, and the graft-derived connectivity is mainly restricted to one of the principal output targets, the globus pallidus, while the substantia nigra is poorly re-innervated.

Grafting in the excitotoxic lesion model has so far been on the replacement of the lost DARPP32 expressing striatal projection neurons. Although the projection neurons are an essential component a fully functional striatal graft likely has to include also the different types of interneurons that are part of the striatal microcircuitry in the intact striatum. The hPSC-derived striatal graft preparations used so far been have been poorly characterized in this regard, and the current protocols likely need to be further developed to achieve a more optimal cellular composition that mimics the neuronal diversity seen in the intact striatum. In contrast to the mDA neuron grafts used in the PD model, the striatal grafts used in the striatal lesion model should be viewed not as simple single neuron replacement, but as a tool to restore a unit of local striatal microcircuitry, composed of both NSPs and regulatory interneurons, that is functionally integrated into the host basal ganglia circuitry.

Complete circuitry reconstruction is more challenging in the PD model since the normal location of the nigral DA neurons is at a relatively large distance from their striatal targets. Full circuitry reconstruction will require that the DA neurons are placed in their normal location, i.e., the ventral midbrain. As reviewed above, in rodent models, the hESC- or fetal-derived human mDA neurons grafted to the nigra can extend their axons along the nigrostriatal pathway and the MFB and establish connections with all normally DA-innervated forebrain targets and receive abundant synaptic inputs from the host. The overall distribution of host-to-graft connections as revealed by rabies tracing matches well the endogenous nigral afferent circuitry, suggesting the possibility for more complete circuitry repair. Whether similar connectivity can be established in the much larger human brain remains to be investigated: the distance between substantia nigra and striatum is about 10-fold larger in humans than in rats, 3–3.5 mm in the rat compared to 3–3.5 cm in the human brain. Whether intranigral grafts are functionally superior to grafts placed in the common intrastriatal location needs also to be clarified. It seems possible that intranigral grafts could have a broader functional impact, e.g., on the recovery of more complex motor behavior and non-motor functions, than the intrastriatal ones, but that is so far not known.

To match the larger size of the human brain, and achieve connectivity over much larger distances, it will be important to find ways to improve the performance of the grafted cells, their survival, integration and growth capacity in particular, as well as their ability to evade the immune/inflammatory response of the host. The survival rate of grafted progenitors or neuroblasts is usually quite low, in the range of 5–20% for grafted fetal DA neurons (Castilho et al., 2000) and a similar range for grafted hESC-derived DA neuron progenitors (Kirkeby et al., 2017a; Niclis et al., 2017). Addition of growth factors (GDNF in particular) or cytoprotective agents, such as lazaroids and caspase inhibitors, can be used to increase this figure about 2-fold, a finding that has justified their use as additives in the cell preparations used in the clinical trials (Castilho et al., 2000).

In more recent years interesting progress has been made in the use of injectable biomaterial scaffolds to improve graft survival and provide a supportive microenvironment for integration and growth of the implanted cells, as well as protection from the host immune response. Such scaffolds can also be loaded with supportive growth factors, as illustrated by the recent studies of Moriarty et al. (2017, 2019a) showing a 4–5-fold increase in DA neuron survival and a 5-fold increase in host striatal reinnervation in 6-OHDA lesioned rats that had received fetal DA neuron grafts enclosed in a GDNF-loaded collagen hydrogel. Also, there is an increasing interest in the role and potential of extracellular matrix proteins, such as laminin and fibronectin, due to their ability to promote the survival, proliferation, and differentiation of fetal and hPSC-derived neural progenitors (Kirkeby et al., 2017b; Somaa et al., 2017; Zhang et al., 2017). For a comprehensive overview of these promising developments, the reader is referred to the recent reviews by Bruggeman et al. (2019) and Moriarty et al. (2019b).

The brain repair field is now entering a new era where the development of new advanced tools for the study of brain connectomics, in combination with cell-specific optogenetic and chemogenetic methods, will provide new possibilities for detailed studies of circuitry dysfunction and cell-based repair. The new tools that have become available during recent years, and the rapidly expanding methods for *in vivo* reprogramming and trans-differentiation, open exciting new opportunities, not only for more refined experimental studies in rodent and primate models but in the longer perspective also for the exploration of circuitry repair in human neurodegenerative disease. Due to its accessibility for experimentation, its attractive anatomical and functional organization, and its clinical relevance, we anticipate that basal ganglia circuitry will remain at the forefront of this development. Exploratory clinical studies using intrastriatal transplants of hPSC-derived mDA neurons in PD patients are now underway (Barker et al., 2017), and similar studies using hPSC-derived striatal neurons in patients with Huntington’s disease are likely to follow in the not too distant future. This development builds on the experiences gained from the clinical studies using fetal VM and GE tissue that have been performed over recent decades (Barker et al., 2013; Lindvall, 2015; Bachoud-Lévi, 2017). Further progress in this field will critically depend on a close interaction between experimental and clinical research where the experience gained from exploratory clinical trials will help to guide and inspire the experimental work, and conversely, where the continued development and refinement of methods and protocols that are investigated experimentally will help to drive the approaches explored in future clinical trials.

## Author Contributions

AB and MP collaborated in writing the article.

## Conflict of Interest

MP is the owner of Parmar Cells AB and co-inventor of two patents in the area of this review (U.S. patent application 15/093,927 owned by Biolamina AB and EP17181588 owned by Miltenyi Biotec). The remaining author declares that the research was conducted in the absence of any commercial or financial relationships that could be construed as a potential conflict of interest.
